# Cutaneous melanoma in Argentina: an analysis of its characteristics and regional differences

**DOI:** 10.3332/ecancer.2020.1017

**Published:** 2020-03-05

**Authors:** Dora Loria, María Graciela Abriata, Federico Santoro, Clara Latorre

**Affiliations:** 1 Argentine Registry of Cutaneous Melanoma, Av Callao 852, C1025 CABA, Argentina; 2 National Ministry of Health (MoH), Av 9 de Julio 1925, C1072 CABA, Argentina; 3 Fellow of Epidemiology, National Ministry of Health, Av 9 de Julio 1925, C1072 CABA, Argentina

**Keywords:** melanoma, Argentina, epidemiology, risk, mortality

## Abstract

**Purpose:**

This study aims to increase the knowledge about the characteristics of cutaneous melanoma in Argentina, their association with the risk of having Breslow ≥1 mm and mortality trends for the period 2002–2017.

**Patients and methods:**

Descriptive statistics and regression analyses were done for 10.199 cases within the Argentine Melanoma Registry in the period 2002–2018. Trends in age-standardised mortality rates (ASMR) were analysed using the Join point Regression Model.

**Results:**

Cases showed lesions mainly located in males’ trunk (37%) and in females’ lower limbs (29%). The level of invasion was higher in males who also showed higher mortality. Cases from the North West and North East regions showed a major risk of Breslow >1 mm and harboured CM in lower limbs more frequently than in other regions. Nearly, 25% of tumours over 2 mm were in cases aged <50 years and 37.6% in patients ≥50 years. In the North West, North East and Patagonia, the frequency of cases in young people was higher than in older people. In 43% of cases, the melanoma subtype was not specified in the report. The number of superficial spreading melanomas, the most common histology, was nearly twice that of Nodular melanomas the following histology in importance (3,403 and 1,754, respectively). Melanoma mortality rates in all Argentine population increased in the elderly. Lower melanoma mortality rates were observed in the North West. In 2007–2017, ASMR decreased significantly in females (average 1.4% p/year) while it increased nonsignificantly in males (0.4% p/year).

The tumours with the worst prognosis were associated with the elderly, males, nodular or acrolentiginous morphologies, residing somewhere other than Centro and Patagonia and with tumors located in the head/neck and legs.

**Conclusion:**

The geographical variations found for melanoma characteristics and their mortality in Argentina, makes it imperative that epidemiological research is continued to avoid generalisations and improve future preventive actions.

## Introduction

The incidence of cutaneous melanoma (CM), the most aggressive skin cancer, has been rising during the past decades in most countries with fair skinned populations with great differences across the globe [1].

In Argentina, Chile, Brazil, Peru and Ecuador the incidence rates were lower than 3 per 100,000 [2, 3].

Five population cancer registries, covering nearly 13% of the total Argentine population, presented incidence data of melanoma for the period 2008–2012, but they are not representing, in a statistical way, the different geographic areas nor the different socioeconomic situations and lack of complete information on tumour stage and histology subtype. The melanoma incidence rates in these five registries were lower than in several other American countries [4].

Argentina is a large and heterogeneous country with 40,117,096 inhabitants (2010) and with big socioeconomic and cultural differences [5]. According to the available demographic data, the population composition of the Argentinean territory is the result of a mixture of European, Native American and Sub-Saharan African populations. As a result of the intermixing, this presents great variations between regions and the type of marker used for studying the genetic admixture. For instance, Argentineans carried 94.1% of European genetic heritage in their Y-chromosomal and 78.5% in the autosomal DNA but their mitochondrial gene pool was mostly of Native American ancestry [6, 7]. African heritage varied between 0.45% and 4.4% according to markers and regions, with the exception of the North West region where a study with blood markers showed a contribution of 14% [8].

It is known that the risk of cutaneous melanoma could be related, in part, to the genetically defined skin colour. In particular, a case-control study of melanoma in Argentina showed that individuals with European grandparents had similar risk factors for CM as individuals in Southern European countries, but people with only Argentine grandparents had a lower CM risk probably due to their darker pigmentation [9].

Currently, the research on melanoma in Argentina is very scarce. To achieve a deep knowledge of melanoma occurrence and its histopathologic characteristics, the Skin Cancer Foundation in Argentina jointly with the Argentine Society of Dermatology founded in the year 2004 the Argentine Registry of Cutaneous Melanoma (RAMC). To our knowledge, this recent analysis of the RAMC cases is the largest melanoma study in Argentina.

Mortality is also a fundamental input for the analysis of situations in health and planning needs for implementing and monitoring health programs.

There are two main objectives of this paper: first, to describe the clinical and histopathologic characteristics of patients with CM and to evaluate possible risk factors related to Breslow thickness, and second, to present melanoma mortality according to regions of residence.

## Materials and methods

Data of residents in Argentina diagnosed with CM between 2002 and 2018 were passively and actively collected by the RAMC. Some details about methods used by the RAMC were previously published [10]. Around 80% of the cases registered in the RAMC were from patients diagnosed / treated in 185 public or private health institutions. The remaining 20% were reported by professionals from their private offices (dermatologists, pathologists, surgeons and oncologists).

Several provincial cancer registries contributed with their cases although not for the complete period: Registry of Mendoza, Entre Rios, Cordoba, Bahia Blanca (In Buenos Aires Province), Santa Fe, Tierra del Fuego, Chaco, Neuquén, Río Negro, Jujuy and the Argentinean Oncopediatric Registry. The Argentinean Network of Hospital Based Cancer Registries (RITA, INC) has also contributed, since 2011, with melanoma cases, coming in 2018 from 40 institutions established in 21 provinces

For the present analysis, demographic variables of cases diagnosed between 2002 and 2018 were: age at diagnosis, sex, and province of residence. As used for national statistics, the provinces were grouped according to geographic area proposals by the Statistics and Health Information Department (DEIS) (see Appendix): Centro, Cuyo North West, North East and Patagonia.

Tumour characteristics included were: topography, histology and tumour behaviour, classified with the International Classification of Diseases for Oncology, ICD-O 3rd E. [11]. Skin invasion was classified according to Clark and Breslow [12, 13].

All statistical tests, performed with STATA package, were two-sided, and *p*-values of 0.05 or lower were considered statistically significant.

Univariate and multivariate logistic regression analysis were performed to identify predictors of higher Breslow level. Odds ratios (OR) and its 95% confidence intervals (95% CI) were computed.

Melanoma mortality data for the period 2002–2017, with cause of death codified with WHO’s International Classification of Diseases, Tenth Revision (ICD-10) was obtained from the DEIS [14]. ASMR were calculated with EPIDAT using world standard population and expressed as deaths per 100,000 persons [15]. Population data for each year was taken from the National Institute of Statistics and Censuses [16].

Joinpoint regression analysis [17] was used to assess ASMR trends and to estimate the annual percentage of change (EAPC) and its 95% CI in the period 2002–2017 and 2007–2017.

## Results

For the 16-year period, 11,321 cases were collected, of which 1,122 were ruled out for not specifying the Argentine province of residence. A total of 10,199 cases with complete information on residence at a sub national level were recorded.

Patient and tumour characteristics are presented in Table 1.

More than 80% of patients in the RAMC cohort had residence in the Centro region. Differences between the distribution of several variables and age group are presented in Table 2.

Site of tumour, histology and tumour thickness were markedly affected by the age. The frequency of lentigo malignant melanoma (LMM) in the elderly age group was nearly three times that in the younger group.

In Table 3, the proportion of melanoma cases with lack of specified data in the main variables is presented. Cuyo, the North East and Patagonia had the higher level of lack of tumour skin site. Cases from Cuyo had also a considerable lack of date of Breslow and histological subtype.

**Figure 1.**
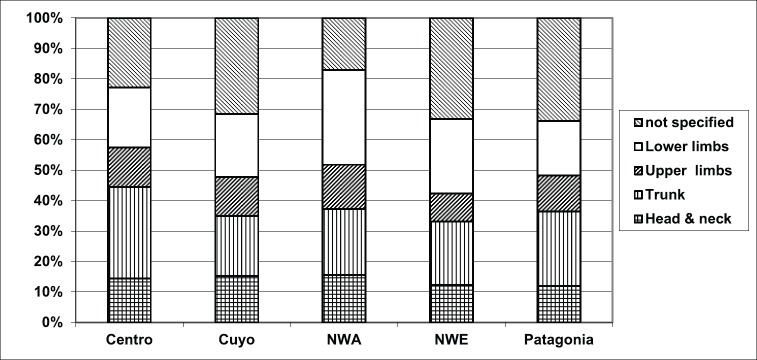
Site of cutaneous melanoma according to Argentine region of residence, RAMC 2002–2018.

**Figure 2.**
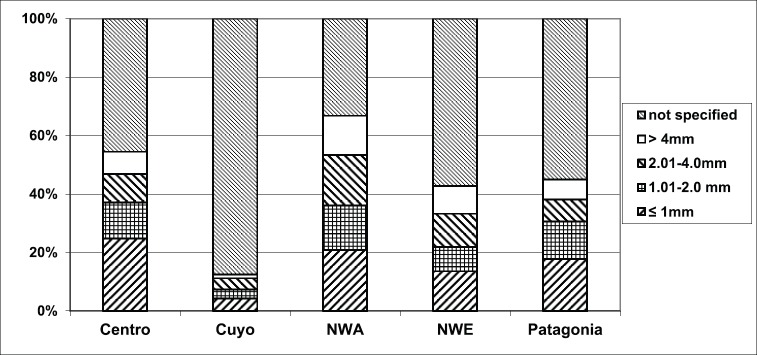
Breslow thickness according to Argentine region of residence, RAMC 2002–2018.

**Figure 3.**
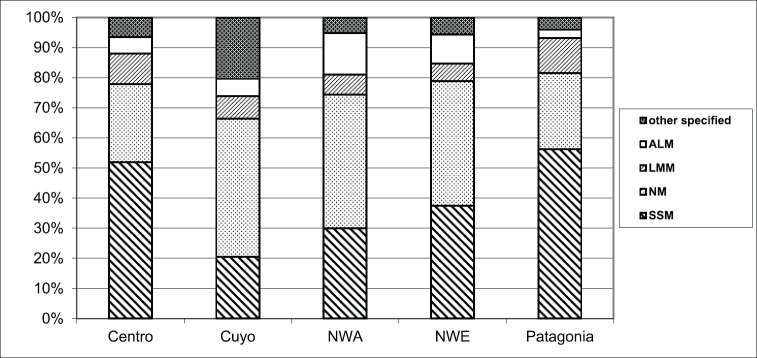
Melanoma subtype according to Argentine region of residence, RAMC 2002–2018.

Excluding cases with unspecified characteristics (Table 2), cases with residence in the North West and North East harbored melanomas in the lower limbs, lesions with Breslow over 2 mm and acrolentiginous subtype (ALM) more frequently than in the other regions (Figures 1–3).

The superficial spreading and nodular lesions appeared in the trunk more frequently than in the other body sites (45.7% and 34.1%). Acrolentiginous melanomas had their highest frequency in the lower limbs (73.2%) and those of the lentigo malignant type in the head and neck (54.2%).

Lesions with more than 4 mm of Breslow deep were mainly nodular (54%, 356 cases) and the 68% (1,364 cases) of lesions with Breslow up to 1 mm was SSM.

The results of the univariate analysis demonstrate that the risk of having lesions with Breslow > 1 mm showed, in comparison with superficial spreading cases, a significant increase for Nodular melanoma cases, presenting a lower increase for the rest of the histological types but lentigo malignant. Considering the Centro region as the basal level, cases from all other regions showed a risk elevation from 20% to 85% (Table 4).

New reference levels were undertaken for the multivariate analysis to present the most robust model among the various possible models. The following independent risk factors were: sex, age, site of primary lesion and region of residence. More than 80% of risk of having Breslow over 1 mm was expected for males over 50 years with residence in the North West or the North East with respect to a basal level composed of young females with residence in the Central region or in Patagonia (Table 5).

Between 2002 and 2017, an annual mean of 480 individuals died due to cutaneous melanoma with an increased mortality rate in elderly patients (Table 6). The number of deaths showed a slow increase in both sexes, with an annual mean of 521 deaths in the period 2013–2017 and with 77% of the occurrence in the Centro region.

**Figure 4.**
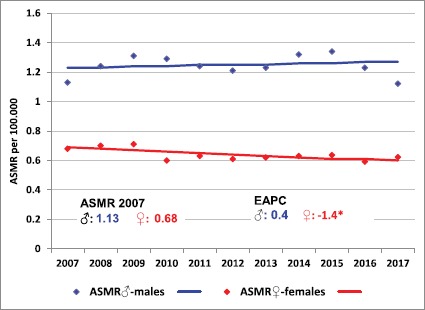
Trends of melanoma mortality rates and estimated annual percent of change by sex, Argentine 2002–2017. (ASMR: age-standaridised mortality rate; EAPC: estimated annual percent of change.)

In each region and in the whole country, ASMR in males was almost double that of females. The behaviour of ASMR differs between sexes and regions. In Centro and Patagonia, both of them decrease in males and females. In Cuyo and the North West, while in females decreasing trends were detected, melanoma mortality rates appeared to be increasing in males. In the North East, the EAPC showed increasing ASMRs for both sexes. Changes in trends were statistically significant in males in the North East and North West (EAPC: 4.2% and 2.2%, respectively) and in women in Cuyo (EAPC: −13.3%), with growing rates for males and decreasing rates for females. In the country, despite the observed rising trends in males and declining trends in females in the two age groups, none of them was statistically significant, with the exception of elderly females (Figure 4 and Table 6)*.*

## Discussion

In a country as extensive as Argentina, with variations in its geography, socioeconomics, quality health systems and population characteristics, the importance of and need for detailed epidemiological studies and taking precautions when making generalisations have become clear.

In Argentina, there are few studies on cutaneous melanoma involving a considerable number of patients. That is why this recent analysis on more than 10,000 cases with its clinical and histological characteristics in the different regions of the country is presented. In parallel, the behaviour of mortality due to this cause was evaluated in order to estimate the situation of having a central mortality data office covering all the country.

Most of the RAMC cases (82%) belonged to the Centro region, as expected, because nearly 65% of the country’s population resides in this region (Appendix).

For the most part, the cases registered without definition of the Argentine province of residence were cases from private pathologists’ offices, which until recently, did not record a complete address of their cases. RAMC is now driving pathologists to collect more details.

Up until today, the RAMC has faced difficulties in getting a more accurate database mainly due to huge health disparities between the regions and complex healthcare fragmentation within the country (subsystems: public, insurance and private) [18]. The large number of health institutions and medical offices makes it difficult for population-based cancer registries to reach a high level of exhaustivity [4].

The lack of completeness in Breslow thickness data, histological type and tumour location is a RAMC weakness to date. An important part of this lack comes from cases reported by the cancer registries. In fact, if we exclude from the present analysis the cases of this origin, there is no detailed information on Breslow, histological type and tumour location in only 26%, 36% and 10% of cases, respectively. The Argentine cancer registries are committed to improving the quality of their data and it is expected that this will also be reflected in an improvement in the quality of the RAMC base with which they collaborate. In addition, active training is driving those who collaborate with their cases and RAMC registrars to reduce missing data.

Notably, a mean of 31.6% of cases with no site specification was also mentioned by five population cancer registries in Argentina and other Central and South American registries for the period 2003–2011 [27]. In the last volume of Cancer Incidence in Five Continents, with data for a more recent period, 2008–2012, the Provincial Cancer Registry of Cordoba showed the highest percentage of unspecified site of tumour (72%), probably due to no retrieved data before being included in the database, whereas the lowest was for the Entre Rios provincial registry (19%) [4].

Gender disparities by several characteristics were observed, suggesting that in Argentina as in many other countries, sex is an important risk for cutaneous melanoma development and prognosis with the risk being higher among males. In line with these results, a significant association between patients age at diagnosis and sex has been previously reported [19, 20]. In the present study, males were, at the time of diagnosis, older than females.

The primary location of melanomas differs between countries and populations. When analysing all RAMC cases together, regardless of gender, 28.6% of the lesions appeared in the trunk, as has been described in Spain [21] and Canada [22], but, for other Latin-American countries, the main location described was the lower limbs [23] or the face [24].

It is well known that for melanoma, the primary site is gender dependent. As in other series of cases [20–22], we reported a predominance of lesions on the trunk in males and on the lower limbs in females. It has been hypothesised that these sex differences could be explained at least in part by a heterogeneous sun exposure patterns with differences in clothing, hair style, occupation, sun-seeking behaviour between males and females [24]. Moreover, the location also differs within ages showing in chronically sun-exposed areas, such as the face, lesions more frequently in the elderly population [1, 24–26]. In our series of cases, head and neck was almost twofold times more frequent in patients’ of age 50 or more versus younger people. Interestingly, for our population, differences in predominant primary site were also observed among patients along the country. In the North West and North East, melanomas in lower limbs appeared more frequently than in Centro, Cuyo or Patagonia. Nevertheless, it’s important to mention that in 24% of cases, the specific site of the tumour was not provided.

Series from some Latin-American countries have shown nodular melanomas as the predominant subtype, followed by superficial spreading, acrolentiginous and lentigo melanomas [28, 29]; or, followed by lentigo [23] or acrolentiginuous melanomas [30]. In this study, as for high CM incidence areas worldwide, the most frequent histology in the country, and for the Centro and Patagonia regions, was the SSM. However, heterogeneous distribution among all histological types appeared for the different regions of the country. For instance, NM was the predominant type for the rest of the country. Acral melanomas, the less common subtype in the country, were more frequent in the North West and East regions with a higher frequency in females than in males. As it happened in Argentina, similar differences in frequency for the predominant subtype within the country were also described for the Brazilian population [28].

When studying histology frequency by age, Argentinean patients with Acroleniginous lesions were diagnosed at older ages. Near the same age at diagnosis for this histological subtype was described in the United States when including blacks, Asian/Pacific Islanders and Hispanic whites’ patients [31]. It is well known that this melanoma subtype is the most common type in the non-white populations. For our region, it was described in a Peruvian cohort of patients, a strong relation between the frequency of ALM and the percentage of people with mixed Spanish and Amerindian origins [32]. Data of the last census in Argentina allowed an estimation of the proportion of people with aboriginal origin, being 1.8% in Centro, 2.0% in Cuyo, 3.4% in the North West, 2.5% in the North East and 6.9% in Patagonia [5]. Moreover, when tested by genetic analysis, a high level of indigenous American ancestry was found in people from the North West region compared to those from the North East or the South of the country, the Patagonia region [6]. Therefore, our findings of a higher frequency of ALM in both Northern regions, is compatible with a greater proportion of indigenous ancestry in these areas. Even though acrolentiginous melanomas represents less than 6% of RAMC cases, it’s important to keep in mind that as we mentioned before, this subtype occurred in the Northern regions more frequently than in other areas. Unfortunately, the RAMC did not collect information about ethnicity, this being a limitation in further analysing racial differences among cases.

A different distribution of CM morphology type was also observed according to patients’ age, with more than threefold times for Lentigo malignant melanomas in individuals over 50. A similar association was also reported in a Colombian study, which showed a predominance of Lentigo lesions mainly located in the head and neck in elderly patients (68%) [26].

In line with results from other studies in several geographic areas [21, 33–35], we observed a significant association between gender and Breslow: males diagnosed with a higher proportion of melanomas with Breslow over 4 mm. These differences between Breslow according to sex and age could be explained by an improvement in self-examination and different habits in young and female individuals [36, 37]. Every year, a Skin Cancer National Awareness Campaign is run in Argentina, with the aim to increase the knowledge about early detection in the population. In these campaigns, more than 70% of all registered participants were females with a mean age of 43-year old, suggesting that females were more aware about primary and secondary prevention strategies (unpublished results).

Within the RAMC database, 50% of the female cases and 48.8% of cases under 50 years had Breslow measure up to 1 mm. The authors agree with other publications that the presence of deeper tumours in older males compared with younger females could be a consequence of the less importance skin changes and controls by males [34]. The result of sex and age as independent risk factors in the multivariate model shown in this study reinforces the hypothesis that females and young individuals devote more attention to their bodies while they are also more aware of health issues.

It is known that prognosis of patients is strongly associated with Breslow thickness [13]. Our results suggest, despite the unfortunate weakness in obtaining more complete data, the importance of projecting personalised preventive actions according to the characteristics of each population to better attract groups at high risk of developing tumours with high Breslow and so worst prognosis. For example, according our results, residents in the North West and East regions must be taken into consideration when running melanoma secondary prevention actions. Moreover, Nodular melanoma was responsible for the highest median Breslow thickness followed by ALM, SSM and LMM. Less favourable tumour thickness for Nodular and acrolentiginous melanomas was also reported in other studies [38, 39].

The situation regarding the mortality data quality is quite different from that of incident cases. Mortality data, disaggregated by age, sex and residence, are available for the whole country and with acceptable quality. Two quality indicators of the death certification have shown that mortality data in Argentina is of good quality. One indicator, the percentage of deaths certified as signs, symptoms and ill-defined conditions, in the period 2003–2017 varied between 5.9% and 9.8% with a medium of 7.6% [40]. Although the provinces with the highest percentage of deaths attributed to these causes are among those of smaller populations, a few deaths from cutaneous melanoma may have been not included in this analysis but most likely without affecting the results presented.

The other indicator is the percentage of ill-defined cancer sites, which in recent years were below 9% [41, 42].

Studies of cutaneous melanoma mortality for specific countries in Latin America are limited. According to the Globocan Project, skin melanoma mortality rates in South America, estimated for 2018, were 0.60/100,000 for females and 1.2/100.000 for males [3]. In Brazil, melanoma mortality differed by regions: 0.26/100,000 in the North; 0.35 in the North East; 0.90 in the South East; 1.74 in the South and 0.64 in the Midwest [33]. Also, in Chile and in Colombia, mortality rates varied along the country [43, 44].

In the present analysis, melanoma mortality rates for the whole country was greater in males than in females with the same reported rate and pattern described in other American countries [43–46].

The decreasing trend of mortality rates in females and the quite stable trend in males observed in the period 2007–2017 could be explained as a better outcome due to a greater search for medical care and a better early detection in Argentinean females. These same causes are the ones that probably explain the different behaviour of mortality in males in Cali, Colombia [47]. Also, a growing trend of mortality in males and a similar behaviour in females was described in Brazil for the years 2000–2014 but the magnitude of such changes was not statistically significant [35]. A different situation was described in Spain, since the mid-90s, where rates have almost levelled off in males and have started to decrease in females and younger age groups, results that could be partially explained by the improvements in diagnosis and treatments in recent decades [46].

Melanoma mortality rates in Argentina showed a heterogeneous geographic pattern, probably by the effect of regional differences in the skin colour prevalence and the accessibility to higher quality health services. Although we identified an increased risk to develop tumours with Breslow >1 mm in patients with residence in both Northern regions, the lower melanoma mortality in the North West could be the result of a lower incidence of CM, due to the high prevalence of indigenous ancestors with darker skin, but there was no data to confirm this hypothesis. However, over the whole period, melanoma mortality in both regions increased, with a near twofold percentage of change by year, specifically for the North East.

Melanoma mortality could increase with age due to several factors, such as higher incidence rates and the diagnosis of thicker lesions in the elderly. Age-specific mortality trends among males and females in the younger age group in Argentina were not statistically significant. Current results indicate that individuals over 50 had a greater risk of mortality than younger ones. For the Southern European population, a significant growth of mortality in middle and older aged individuals was described [48].

Cancer incidence rates for the whole country were estimated [3, 4] but we suggest that those results must be taken with caution because, as stated previously, Argentina is a very heterogenic country and these estimates of incidence rates for the country did not represent the different areas.

The RAMC database is neither an exhaustive collection of cutaneous melanoma cases nor a probabilistic sample and, according to this lack in exhaustiveness, it was not possible to calculate incidence rates for the whole country. As for many retrospectives studies, a limitation of our work was the incomplete data about some characteristics at diagnosis that could affect some results. Moreover, a lack of complete information on tumour stage and histology subtype in incidence data has been previously reported from most Latin American cancer registries [4].

Regarding mortality results, and given the acceptable figures of quality indicators of death data, it is unlikely that some of the observed differences could be entirely due to intrinsic data problems.

## Conclusion

It is important to emphasise the need to continue to promote these types of studies to learn more about the impact of melanoma’s burden of disease in our population, and the risk factors associated with developing deep tumours throughout the entire country. Results from these types of studies may be useful to improve primary and secondary prevention campaigns and early detection of tumours in countries such as Argentina.

## Funding

The Argentine Registry of Cutaneous Melanoma activities were financially supported through a grant from the “Fundación del Cáncer de Piel, Argentina”. At the same time, this foundation received a non-restricted grant from La Roche Posay and Vichy Laboratories to finance their activities in Argentina.

## Conflicts of interest

All authors certify that they have no affiliations with or involvement in any organisation or entity with any financial interest or non-financial interest in the subject matter or materials discussed in this manuscript.

## Figures and Tables

**Table 1.** Cases characteristics and correlations beteen patient sex and clinic pathological variables (RAMC, 2002–2018).

Variables	Total (%)	Women (%)	Men (%)	p*
**Cases**	10,199	4,982 (48.9)	5,217 (51.1)	
Age mean, years (SD)	57.4 (17.5)	55.8 (18.2)	59.0 (16.6)	
**Age (years) (%)**				
<50	3,251 (31.9)	1,819 (36.5)	1,432 (27.5)	0.000
≥ 50	6,808 (66.7)	3,091 (62.0)	3,717 (71.2)	
n.s.	140 (1.4)	72 (1.5)	68 (1.3)	
**Tumor behaviour**				
In situ (TIS)	1,241 (12.2)	607 (12.2)	634 (12.1)	
Invasive	4,634 (45.4)	2,226 (44.7)	2,408 (46.2)	
n.s.	4.342 (42.4)	2,149 (43.1)	2,175 (41.7)	
**Anatomic site, *n* (%)**				
Head & neck	1,459 (14.3)	655 (13.2)	804 (15.4)	
Trunk	2,913 (28.6)	1,001 (20.1)	1,912 (36.6)	
Upper extremities	1,307 (12.8)	695 (14.0)	612 (11.7)	
Lower extremities	2,060 (20.2)	1,439 (28.9)	621 (11.9)	
n.s.	2,460 (24.1)	1,192 (23.8)	1,268 (24.4)	
**Histology, n (%)**				
Superficial spreading	2,855 (28.0)	1,426 (28.6)	1,429 (27.5)	0.000
Nodular	689 (15.8)	921 (13.8)	1,610 (17.7)	
Lentigo maligna	574 (5.6)	287 (5.8)	287 (5.5)	
Acral lentiginous	332 (3.3)	209 (4.2)	123 (2.4)	
Other specified	402 (3.9)	188 (3.8)	214 (4.1)	
n.s.	4,426 (43.4)	2,183 (43.8)	2,243 (42.8)	
**Breslow depth**				
Median in mm (range)	1.23 (0.93-79.0)	1.1 (0.03-65.0)	1.5 (0.03-70.0)	0.000
Depth range, *n* (%)				
≤1 mm	2,006 (22.6)	1,007 (24.6)	929 (20.3)	
1.01–2.0 mm	1,045 (11.5)	482 (11.0)	563 (12.3)	
2.01–4.0 mm	858 (9.5)	360 (8.2)	498 (10.9)	
>4.0 mm	660 (7.4)	282 (6.5)	378 (8.3)	
n.s.	4,389 (49.0)	2,174 (49.7)	2,215 (48.2)	
**Clark level, *n* (%)**				
I	1,216 (11.9)	599 (12.0)	617 (11.8)	0.001
II	1,237 (12.1)	652 (13.1)	585 (11.2)	
III	1,537 (15.1)	721 (14.5)	816 (15.6)	
IV	1,313 (12.9)	582 (11.7)	731 (14.0)	
V	256 (2.5)	127 (2.6)	129 (2.5)	
n.s.	4,640 (45.5)	2,301 (46.1)	2,339 (44.9)	
**Ulceration, *n* (%)**				
Present	1,292 (14.7)	566 (13.1)	726 (16.1)	0.000
Non present	2,656 (30.1)	1,324 (30.7)	1,332 (29.6)	
n.s.	4.866 (55.2)	2.419 (56.2)	2.447 (54.3)	
**Region of residence**				
Centro	8,347 (81.8)	4,050 (81.2)	4.297 (82.3)	0.158
Cuyo	705 (6.9)	357 (7.2)	348 (6.7)	
NORTH WEST	380 (3.7)	207 (4.2)	173 (3.3)	
NORTH EAST	253 (2.5)	125 (2.5)	128 (2.5)	
Patagonia	514 (5.1)	243 (4.9)	271 (5.2)	

* *p*-value from testing the difference between men and women. n.s: not specified/no data.

**Table 2.** Characteristics of cutaneous melanoma cases by age group (RAMC, 2002–2018).

Characteristic	<50 years	≥ 50 years	p value *
**Anatomic site, *n* (%)**			
Head & neck	297 (9.1)	1,149 (16.9)	0.000
Trunk	1,058 (32.6)	1,818 (26.7)	
Upper extremities	430 (13.2)	864 (12.7)	
Lower extremities	717 (22.1)	1,323 (19.4)	
n.s.	749 (23.0)	1,654 (24.3)	
**Histology, *n* (%)**			
Superficial spreading	1,100 (33.8)	1,711 (25.1)	0.000
Nodular	471 (14.5)	1,114 (16.4)	
Lentigo maligna	70 (2.2)	500 (7.3)	
Acral lentiginous	62 (1.9)	267 (3.9)	
Other specified	114 (3.5)	280 (4.1)	
n.s.	1,434 (44.1)	2,936 (43.2)	
**Breslow depth, *n* (%)**			
≤1 mm	789 (27.3)	1,190 (20.0)	0.000
1.01–2.0 mm	373 (12.9)	657 (11.1)	
2.01–4.0 mm	235 (8.1)	607 (10.2)	
> 4.0 mm	179 (6.2)	472 (7.9)	
n.s.	1,317 (45.5)	3,020 (50.8)	
**Clark level, *n* (%)**			
I	350 (10.8)	845 (12.4)	0.000
II	489 (15.0)	729 (10.7)	
III	544 (16.7)	972 (14.3)	
IV	366 (11.3)	933 (13.7)	
V	71 (2.2)	180 (2.6)	
n.s.	1,431 (44.0)	3,149 (46.3)	
**Region of residence**			
Centro	2,588 (79.6)	5,651 (83.0)	0.000
Cuyo	198 (6.1)	507 (7.4)	
North West	132 (4.1)	236 (3.5)	
North East	111 (3.4)	141 (2.1)	
Patagonia	222 (6.8)	273 (4.0)	

* *p*-value from testing the difference between age groups. n.s: not specified/no data.

**Table 3.** Percentage of anatomic site, Breslow depth and histology subtype without a specific data by region of residence (RAMC, 2002–2018).

Geographic region	Anatomic site	Breslow depth	Histology
Centro	22.9	45.5	41.7
Cuyo	31.6	87.5	60.4
North West	17.1	33.2	39.5
North East	33.2	57.2	58.9
Patagonia	33.8	55.0	43.2

**Table 4.** Univariate logistic regression analysis of CM risk factors for Breslow thickness (RAMC, 2002–2018).

Variable	OR ( 95% CI )*	p-value**
**Sex**		
Women &	1.00	0.000
Men	1.49 (1.32-1.67)	
**Age, years**		
<50 &	1.00	0.000
≥ 50	1.47 (1.30- 1.66)	
**Anatomic site, *n* (%)**		
Trunk &	1.00	
Head & neck	1.27 (1.05-1.53)	0.014
Arm	1.00 (0.84-1.19)	0.9889
Leg	1.40 (1.21-1.62)	0.000
n.s.	1.55 (1.16-2.07)	0.003
**Histology, *n* (%)**		
SSM &	1.00	
NM	17.88 (14.31-22.35)	0.000
LM	0,62 (0,42-0.92)	0.017
ALM	4.92 (3.60-6.73)	0.000
Other specified	4.73 (3.35-6.70)	0.000
n.s.	2.54 (2.16-3.00)	0.000
**Region of residence**		
Centro &	1.00	
Cuyo	1.70 (1.08-2.68)	0.022
North West	1.84 (1.39-2.44)	0.000
North East	1.80 (1.18-2.73)	0.006
Patagonia	1.67 (0.95-1.68)	0.107

* Odds ratio and 95% Confidence interval

** *p* value; & reference category; n.s.: not specified/no data.

**Table 5.** Multivariate logistic regression analysis of CM risk factors for Breslow thickness (RAMC, 2002–2018).

Variable	OR (95% CI)*	p-value**
**Sex**		
Women	Reference &	
Men	1.61 (1.41–1.83)	0.000
**Age , years**		
<50	Reference &	
≥ 50	1.40 (1.23–1.59)	0.000
n.s.	1.38 (0.83–2.30)	0.212
**Anatomic site, *N* (%)**		
Trunk and upper extremities	Reference &	
Head & neck	1.19 (0.99–1.44)	0.065
Lower extremities	1.62 (1.40–1.88)	0.000
n.s.	1.56 (1.16–2.08)	0.003
**Region of residence**		
Centro & Patagonia	Reference &	
Cuyo	1.66 (0.98–2.80)	0.057
North West	1.80 (1.34–2.42)	0.000
North East	1.80 (1.17–2.76)	0.007

* Odds ratio and 95% confidence interval

** *p* value; & reference category; n.s.: not specified/no data.

**Table 6.** Trends of melanoma age standardised mortality rates by sex in each geographic regions and by sex and age groups in the country Argentina, 2002–2017.

Region	ASMR* 2002	Trends		ASMR* 2017
		Period	EAPC (95% CI) **	
**Argentina**				
Male	1.24	2002–2015***	0.9 (0.2; 1.7)	1.12
		2015–2017	−7.3 (−18.8; 5.9)	
Female	0.60	2002–2017***	−1.2 (−1.7; −0.5)	0.62
**Centro**				
Male	1.38	2002–2017	−0.2 (−1.1; 0.7)	1.21
Female	0.67	2002–2017***	−1.0 (−1.9; 0.0)	0.73
**Cuyo**				
Male	1.00	2002–2017	2.3 (−0.5; 5.3)	0.64
Female	0.43	2002–2012	1.4 (−1.1; 4.0)	0.34
		2012–2017***	−13.3 (−20.4; −5.5)	
**North West**				
Male	0.60	2002–2017***	2.2 (0.2; 4.3)	0.66
Female	0.34	2002–2017	−1.7 (−5.1; 1.8)	0.40
**North East**				
Male	1.08	2002–2017***	4.2 (1.3; 7.3)	1.48
Female	0.54	2002–2017	3.3 (−0.4; 7.2)	0.67
**Patagonia**				
Male	1.18	2002–2017	−0.7 (−4.0; 2.6)	1.13
Female	0.65	2002–2017	−0.9 (−4.1; 2.4)	0.16
**Argentina**				
Male				
<50 years	0.26	2002–2017	0.6 (−0.8; 2.1)	0.26
≥ 50 years	4.91	2002–2017	0.7 (−0.1; 1.6)	5.28
Female				
<50 years	0.22	2002–2017	−1.9 (−4.2; 0.4)	0.19
≥ 50 years	3.04	2002–2017***	−0.6 (−1.0; 1.0)	2.99

* Age standardise mortality rate

** estimated annual percentage of change and 95% confidence interval

*** time period with EAPC statistically significative.

## Appendix

Argentine population and percentage of regions and provinces in the country, 2010 [5].

**Table:**

Argentina		40.788.453	(100%)
**Region**	**Province**	**Population**	**(%)**
**Centro**		**26.631.929**	**(66%)**
	CABA	3.028.481	(8%)
	Buenos Aires	15.716.942	(39%)
	Córdoba	3.373.025	(8%)
	Entre Ríos	1.255.574	(3%)
	Santa Fe	3.257.907	(8%)
**North East**		**3.762.653**	**(8%)**
	Chaco	1.080.017	(2%)
	Corrientes	1.017.731	(2%)
	Formosa	551.626	(1%)
	Misiones	1.113.279	(3%)
**North West**		**4.668.771**	**(12%)**
	Catamarca	377.676	(1%)
	Jujuy	683.513	(2%)
	Salta	1.239.111	(3%)
	Santiago del Estero	879.246	(2%)
	Tucumán	1.489.225	(4%)
**Cuyo**		**3.257.339**	**(8%)**
	La Rioja	342.582	(1%)
	Mendoza	1.774.737	(4%)
	San Juan	696.076	(2%)
	San Luis	443.944	(1%)
**Patagonia**		**2.467.761**	**(6%)**
	Chubut	513.433	(1%)
	La Pampa	327.028	(1%)
	Neuquén	571.910	(1%)
	Río Negro	648.277	(2%)
	Santa Cruz	275.452	(1%)
	Tierra del Fuego	131.661	(0%)
